# Posterolateral elbow dislocation with entrapment of the medial epicondyle in children: a case report

**DOI:** 10.4076/1757-1626-2-6603

**Published:** 2009-07-27

**Authors:** Juan Rodríguez Martín, Juan Pretell Mazzini

**Affiliations:** Department of Orthopaedic Surgery, 12 Octubre HospitalAv. Andalucia s/n, Madrid, 28045Spain

## Abstract

**Introduction:**

Elbow dislocations in children are uncommon injuries. Dislocations with associated fractures or so-called complex dislocations of the elbow can be challenging to diagnose and treat.

**Case presentation:**

A 14-year-old male had a posterolateral elbow dislocation after a fall. Closed reduction with traction was performed. Radiographs after initial reduction showed a fragment entrapped into the humero-cubital joint. Computerized tomography scan showed the fragment belonging to the medial epicondyle. Open reduction and internal fixation with a 3.0 millimeter cannulated screw was performed, with restoring of the normal function of the elbow at final follow up.

**Conclusion:**

Elbow dislocations in children can be associated with bone lesions. These injuries must be suspected to avoid misleading diagnosis and achieve good results.

## Introduction

Traumatic dislocation of the elbow is rare in children with an incidence of only 3% to 6% of all elbow injuries. Pure dislocations are uncommon and radiographs must be evaluated carefully for associated injuries, which can include fractures or avulsions of the medial epicondyle, coronoid process, radial head, trochlea and lateral condyle, or disruption of the proximal radio-ulnar joint [1-3].

## Case presentation

A 14-year-old Caucasian Spanish male, right hand dominant presented to the emergency department after falling on his left hand with elbow extension. On examination there was gross deformity and swelling on his elbow. There were no external wounds and no neurovascular involvement. No abnormalities were found in ipsilateral shoulder and wrist joints.

**Figure 1.**
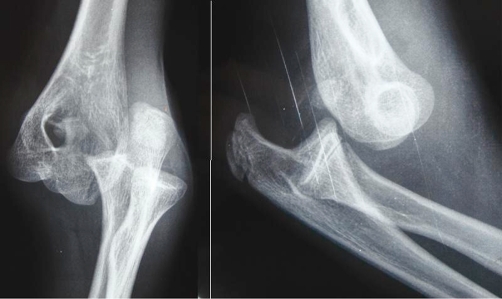
Radiographs showing a posterolateral dislocation of the elbow. **(A)** Anteroposterior view. **(B)** Lateral view.

**Figure 2.**
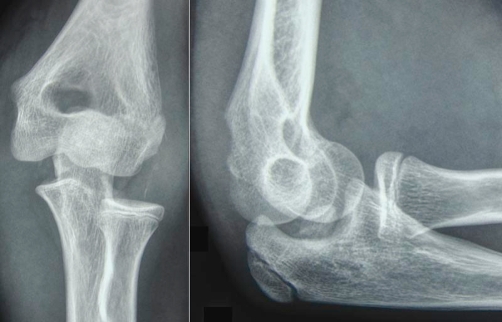
Radiographs performed after closed reduction. **(A)** Anteroposterior view showing incongruity of the elbow joint. **(B)** Lateral view. A bone fragment is clearly identified into the joint.

**Figure 3.**
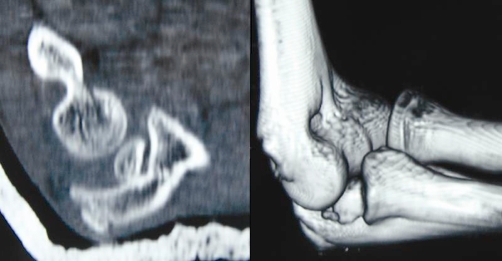
CT scan images. **(A)** Intraarticular fragment located into the humerocubital joint. **(B)** 3D reconstruction image. The fragment belonged to the medial epicondyle.

Plain radiographs of the elbow revealed a posterolateral dislocation of the elbow (Figure 1A & 1B). Closed reduction with traction in prone position was performed immediately. After initial reduction, the patient was neurovascular intact, but referred no pain improvement. Examination revealed no possibility of passive flexion or extension through the elbow joint and valgus instability. Post-reduction radiographs showed incongruity of the joint with an entrapment fragment into the humero-cubital joint (Figure 2A & 2B). A CT scan was performed revealing the intraarticular fragment belonging to the medial epicondyle (Figure 3A & 3B).

**Figure 4.**
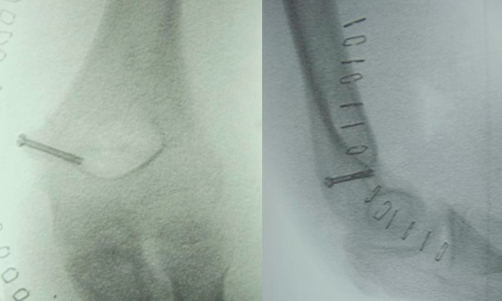
Intraoperative images after reconstruction. **(A)** Anteroposterior view. **(B)** Lateral view.

After written consent from the parents, surgery was performed. The patient was placed in supine position with the injured elbow on a hand table under general anaesthesia, with a tourniquet and prophylactic antibiotic administration. A medial approach to the elbow joint was performed, and the ulnar nerve was identified and protected. A tear of the origin of the wrist flexors on the medial epicondyle was observed. The epicondylar fragment was removed from the joint and fixed anatomically into its origin with a 3.0 mm diameter cannulated lag screw (Synthes, Solothurn, Switzerland), under fluoroscopy control (Figures 4A & 4B). Rigid fixation was achieved. The joint was inspected to be free of any small fragments of bone. Intraoperative testing revealed stability of the joint in the entire range of motion and varus and valgus stress. Postoperatively, a posterior above-elbow splint with the elbow joint in 90º of flexion and neutral rotation was applied for 7 days followed by early motion and a physiotherapy program to improve the muscle strength. At 3 months of follow up the patient had almost full range of motion with only a lack of 5 degrees of extension and slight atrophy of biceps and triceps muscles. At 6 months he restored full range of motion and muscle strength.

At final follow up at one year postoperatively there was full range of motion with no pain or instability, and no valgus or varus deformity was observed.

## Discussion

Elbow dislocations in children are less frequent than in adults, and majority of the dislocations are toward the posterolateral. Closed reduction and immobilization are indicated for the treatment of isolated elbow dislocations. However, if there is an associated fracture, the treatment is determined depending on the position and displacement of the fracture fragment, and whether it is intraarticular or not following the reduction. Medial epicondyle fracture can be found after a posterolateral dislocation in children [2]. In adults usually the medial collateral ligaments torns after the dislocation, but in children this ligament can produce a displaced Salter-Harris type I fracture of the medial epicondyle. The epicondyle is small and when displaced it is difficult to see on routine radiographs; it may be overlapped by the distal humeral metaphysis or confused with the ossification centre of he trochlea or olecranon. In the case presented, the fracture was misleading initially and was identified after the initial reduction.

The management of this injury is controversial. Some authors have advocated open reduction and internal fixation in all cases to prevent instability and non union [4]. Others have considered surgery for the entrapped or displaced epicondyle to restore stability to the elbow [1,5,6]. Other surgical indication is the displacement of the fragment. Hines et al. [7] suggested surgical treatment for medial epicondyle fractures with a displacement over 2 mm whereas Fowles et al. [8] reported good results with conservative treatment even in the presence of displacement. Kobayashi et al. [9] highlighted the significance of the size as well as the degree of displacement of the fragment in epicondyle fractures, and suggested that conservative treatment is indicated for patients in whom the maximum diameter of bone fragment is 13 mm or less or the displacement of the bone fragment is 9 mm or less.

Delay in diagnosis of this injury can occur because of lack of interpretation of the radiographs. There should be a high index of suspicion, with good clinical examination and meticulous assessment of the radiographs. Elbow dislocations in children should ideally be reduced under general anaesthesia and radiological control to avoid delay in accurate diagnosis.

## Conclusion

Elbow dislocations in children are uncommon. Associated injuries must be suspected. The dislocation should ideally be reduced under general anaesthesia and radiological control to avoid delay in accurate diagnosis.

## Abbreviation

CT: Computerized Tomography
